# Rapid Qualitative Approaches in Pandemic Research: Protocol for an Exploratory Qualitative Multimethod Study (VERDIQual) on Mpox in Italy, Nigeria, Thailand, and the United Kingdom

**DOI:** 10.2196/77321

**Published:** 2026-01-15

**Authors:** Chinye Osa-Afiana, Marthe Le Prevost, Emily Jay Nicholls, Davide Bilardi, Thomas E Guadamuz, Esekwe E Soje-Amadosi, Grace I Adebisi, Chibueze Adirieje, Adeyosola A Adetunji, Karima Yusufu, T Charles Witzel, Worawalan Waratworawan, Nattharat Samoh, Tom May, Sarah Denford, Willian Gomes, Uzodinma Adirieje, Shema Tariq, Nadia Adjoa Sam-Agudu

**Affiliations:** 1 International Research Center of Excellence Institute of Human Virology Nigeria Abuja Nigeria; 2 MRC Clinical Trials Unit University College London London United Kingdom; 3 Institute for Global Health University College London London United Kingdom; 4 Fondazione Penta Padua Italy; 5 Nuffield Department of Primary Care Health Sciences University of Oxford Oxford, England United Kingdom; 6 Mahidol Center for Health, Behavior and Society Faculty of Tropical Medicine Mahidol University Nakorn Pathom Thailand; 7 Pediatric and Adolescent ART Unit Department of Prevention, Care and Treatment Institute of Human Virology Nigeria Abuja Nigeria; 8 Faculty of Social Sciences and Humanities Department of Society and Health Mahidol University Nakorn Pathom Thailand; 9 NIHR Health Protection Research Unit in Evaluation and Behavioural Science University of Bristol Bristol United Kingdom; 10 Population Health Sciences Bristol Medical School University of Bristol Bristol United Kingdom; 11 European AIDS Treatment Group Lisbon Portugal; 12 Afrihealth Optonet Association Abuja Nigeria; 13 Mortimer Market Centre, Central and Northwest London NHS Foundation Trust London, England United Kingdom; 14 Department of Paediatrics and Child Health School of Medical Sciences University of Cape Coast Cape Coast, Central Ghana; 15 Global Pediatrics Program and Division of Pediatric Infectious Diseases Department of Pediatrics University of Minnesota Medical School Minneapolis, MN United States

**Keywords:** pandemics, mpox, monkeypox, global health, qualitative research

## Abstract

**Background:**

Recent mpox outbreaks have underscored significant gaps in global preparedness for emerging and re-emerging infections. These outbreaks have disproportionately affected vulnerable and marginalized populations, exposing the weaknesses of health systems, particularly in resource-limited settings. The global spread of mpox beyond endemic African countries in 2022 and the emergence of a new Clade Ib in 2024 emphasize the pressing need for comprehensive and context-specific public health responses. We outline the protocol for an innovative multimethod qualitative study (VERDIQual [SARS-CoV-2 (and Mpox) Variants Evaluation in Pregnancy and Paediatrics Cohorts Qualitative Study]). This study is being conducted across 4 different countries and settings—Italy, Nigeria, Thailand, and the United Kingdom.

**Objective:**

VERDIQual uses multiple qualitative methods to explore the lived experiences of different populations with mpox and frontline health care workers in endemic and nonendemic settings. With this approach, we aim to identify missed and new opportunities for effective public health messaging on prevention and treatment in this and future pandemics.

**Methods:**

VERDIQual’s flexible, multimethod approach integrates content analysis of news and social media, focus group discussions, semistructured interviews, and participatory photography. We apply intersectionality theory to capture perspectives from a diverse range of participants, including gay, bisexual, and other men who have sex with men, pregnant women, adolescents with mpox, and frontline health care workers providing mpox services. Data will be collected and analyzed using the rapid assessment procedure approach, enabling real-time data synthesis to provide timely and contextually relevant insights. We use a standardized approach to data integration at the interpretation stage, summarizing findings in a multimethod integration matrix to visualize and synthesize data by objective, method, and site.

**Results:**

Data collection tools, including focus group discussions and semistructured interview topic guides, have been developed in collaboration with local community advisory boards. Data collection was completed by October 31, 2025, and analysis is ongoing. Teams across the 4 countries have identified media houses and social media platforms for inclusion in the news and social media analysis. Study results will be disseminated through peer-reviewed journals, scientific conferences, policy briefs, and stakeholder workshops and community events, with a focus on informing equitable and inclusive future public health responses to re-emerging infections.

**Conclusions:**

We expect our principal findings to be applicable to a range of settings. Our use of intersectionality theory will also facilitate considerations for intersecting identities and characteristics in equity-centered pandemic responses. Ultimately, we expect VERDIQual to inform pandemic preparedness, inclusive of people with stigmatized and vulnerable characteristics or identities.

**International Registered Report Identifier (IRRID):**

DERR1-10.2196/77321

## Introduction

The recent COVID-19 and mpox pandemics have brought attention to global preparedness for emerging and re-emerging infectious disease outbreaks [1-3]. Certain populations have borne the brunt of shortfalls in health systems’ infrastructure and preparedness. For example, children and pregnant women have experienced exclusion from intervention studies, delayed vaccination, delayed or inadequate treatment options, and poor inclusion in public health policy [4]. Furthermore, marginalized groups such as those who are racially or gender and sexual minoritized are often at greatest risk of infection and poorer clinical outcomes [5,6]. The needs of such key populations should be considered and promptly addressed at all stages of pandemics, including prepandemic planning and postpandemic surveillance.

Since the first reported case of human mpox in the Democratic Republic of the Congo in 1970 [7], mpox case reports have largely been limited to endemic countries in West and Central Africa [7]. The rare cases reported in nonendemic countries were linked to travel to or exposure to animals transported from an endemic African country [8]. This changed in May 2022, when a large number of mpox cases were first reported in European countries, later spreading outside Europe [8]. The World Health Organization (WHO) declared mpox a Public Health Emergency of International Concern in July 2022, in response to mpox being reported in more than 70 countries [8]. In May 2023, the WHO announced the end of mpox’s status as a Public Health Emergency of International Concern due to sustained reduction in incident cases [9]. However, in 2024, a new outbreak of mpox was identified in Central Africa, attributed to the emergence of a distinct Clade Ib variant [9,10].

Over 85% of cases in the global 2022 mpox outbreak were among gay, bisexual, and other men who have sex with men; also, HIV coinfection was reported in over 50% of global mpox cases [9]. The ongoing Clade Ib outbreak has a different epidemiological profile, having emerged in eastern Democratic Republic of the Congo in 2024 [10]. It predominantly affects nonendemic areas within the Democratic Republic of the Congo and neighboring countries, with transmission occurring across different biological sexes (male and female) and sexual behavior. Infection is largely influenced by factors such as individual exposure risk and immune status, with children and pregnant women being at greatest risk due to lower immunity and increased vulnerability to poorer health outcomes [9].

Mpox epidemiology in African countries contrasts with global data. Reports from the WHO African region indicate a general population profile, with endemic West and Central African countries bearing over 99% of the case burden [8,9]. There is a paucity of robust data on mpox incidence and mortality in children [8,9], pregnant women, and groups at high risk (such as people living with HIV), all of whom are the most affected during infectious disease outbreaks [8].

Like other infectious disease outbreaks, the recent mpox outbreaks are complex biosocial phenomena, disproportionately impacting marginalized and vulnerable groups and exposing inadequacies in pandemic responses. The HIV [11] and COVID-19 pandemics [12] have highlighted the central importance of considering social contexts in planning and implementing outbreak responses.

Qualitative research plays a crucial role in understanding the interplay of human behavior, social dynamics, and public health responses. It helps uncover the social, political, and cultural factors that shape experiences and influence the implementation of health interventions. For example, qualitative data can provide insights into decision-making about vaccination and perceptions of public health messaging [13,14]. This allows for public health policy and interventions to be tailored to specific populations while minimizing potential harms such as stigma. Furthermore, such approaches generate rich insights into lived experiences, yielding contextual understanding of the multidimensional impacts of pandemics [15].

Epidemiological data indicate that children and young male adults have been the most affected in Africa [16]. Beyond intense bias against sexual and gender minorities, mpox stigma in these settings is shaped by fear of contagion, the visibility of skin lesions, misinformation, and association of infection with poverty or perceived promiscuity [17,18]. These drivers of stigma intersect with systemic issues such as social inequality and weak health infrastructure [19-21]. Studies conducted among gay, bisexual, and other men who have sex with men with mpox in Australia, China, and the United Kingdom have found that stigma was central to their experiences and was often exacerbated at health facilities [22-25]. Other studies have focused on the role of news and social media (eg, Twitter [subsequently rebranded X] and Reddit) in spreading or countering mis- or disinformation [26], with less data available to understand information-sharing on newer social media platforms such as TikTok and Instagram.

There has been limited qualitative research on mpox, especially in endemic African countries. Studies have predominantly focused on gay, bisexual, and other men who have sex with men and high-income settings, with a lack of data on experiences in endemic African regions and other resource-limited settings [14]. In addition, there is a relative neglect of how multiple experiences of marginalization intersect to shape experiences of mpox and prevention. Furthermore, limited community engagement in research design and implementation results in studies that insufficiently address community priorities and needs. We aim to directly address these gaps through our expanded geographic and population scope, intersectionality-informed analyses, strong patient and public involvement, and inclusive and innovative methodologies, drawing from data that have been underused in mpox research.

The European Union–funded VERDI (SARS-CoV-2 (and Mpox) Variants Evaluation in Pregnancy and Paediatrics Cohorts) consortium is a global partnership between 30 centers of excellence in Europe, the United States, Africa, the Caribbean, the Middle East, and Southeast Asia [27]. VERDI prioritizes children, pregnant women, and vulnerable groups in current and future pandemic research. The consortium also aims to inform preparedness and public health responses for future infectious disease outbreaks in these populations, drawing upon recent experiences with COVID-19 and mpox. One of VERDI’s projects is a collaborative multicountry qualitative study on mpox (VERDIQual [SARS-CoV-2 (and Mpox) Variants Evaluation in Pregnancy and Paediatrics Cohorts Qualitative Study]), which we describe in this paper [27].

VERDIQual supports the broader goals of the VERDI consortium by providing a contextual understanding of the experiences and needs of diverse populations in different geographical settings during the 2022 global mpox outbreak. Our findings are expected to inform more equitable access to relevant interventions, reduce stigma, and improve public health messaging and the research response for ongoing and future pandemics. Specific objectives are (1) to understand the lived experiences of individuals who acquired or were otherwise affected by mpox, (2) to identify missed and new opportunities for effective public health messaging on prevention and treatment, and (3) to develop capacity and establish methods for collaborative rapid qualitative research in response to outbreaks of emerging and re-emerging infections.

## Methods

### Overall Study Design

This exploratory multimethod, multisite qualitative study comprises content analysis of news and social media, focus group discussions (FGDs), semistructured interviews (SSIs), and participatory photography and uses rapid qualitative analysis approaches. The study is being conducted across 4 countries: Italy, Nigeria, Thailand, and the United Kingdom. Study teams in each country have received rigorous training and are experienced in each of these methods. Study countries were purposely selected based on the number of reported mpox cases, geographical spread (Africa, Asia, and Europe), diversity of mpox epidemiology (sexual and gender minority vs general populations in nonendemic vs endemic settings), and resources (high-income [United Kingdom and Italy] vs low- and middle-income countries [Nigeria and Thailand]). The methods used in each country will vary according to resources and feasibility (Table 1). For example, participatory photography was considered feasible and acceptable for target study participants with recently acquired mpox in Thailand; however, this was a less familiar method in Nigeria, and there was a concern for exacerbation of stigma or self-stigma and low acceptability among people with mpox and their communities. The UK team could not conduct SSIs among people with recently acquired mpox due to the significant decline in cases by late 2022. The flexibility for different country teams to use different methods across reflects VERDIQual’s commitment to country team autonomy and the accommodation of local realities and norms with respect to the feasibility and acceptability of the different research approaches. We present our study design using the COREQ (Consolidated Criteria for Reporting Qualitative Research) checklist [28] (Multimedia Appendix 1).

**Table 1:** VERDIQual^a^ data collection methods by country.

	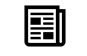 News media	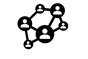 Social media	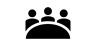 Focus groups	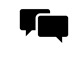 Interviews	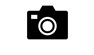 Photography
Italy	✓				
Nigeria	✓	✓	✓	✓	
Thailand		✓	✓	✓	✓
United Kingdom	✓	✓	✓		

^a^VERDIQual: SARS-CoV-2 (and Mpox) Variants Evaluation in Pregnancy and Paediatrics Cohorts Qualitative Study.

### Theoretical Framework

This study is informed by intersectionality theory, a framework for understanding how multiple social categories, such as race, gender, sex, sexuality, and geographic residence, interact to shape unique experiences of privilege and oppression that cannot be captured by considering each category independently [29]. The principles of intersectionality theory will be embedded throughout the life cycle of the study. We are specifically interested in how multiple social identities and structural forces combine to influence risk and experiences of mpox, resisting reducing populations to homogenous groups. We will do this by sampling participants with diverse characteristics and identities in terms of race, gender (being a man or woman), sex (male or female), sexuality (heterosexual, lesbian, gay, bisexual, or transgender), and HIV status. For example, in the United Kingdom, we have collaborated with a charity advocating for the sexual health and well-being needs of racially minoritized gay, bisexual, and other men who have sex with men. In Nigeria, we have partnered with an organization with extensive nationwide experience in community-based research and community-level health promotion. In analyses, we will use coding frameworks that are sensitive to co-occurring and overlapping forms of disadvantage and privilege, critically reflecting on how multiple identities interact to produce mpox-related stigma. Finally, comparative analyses will be conducted across all sites to yield insights into geographic variation in experiences of and attitudes to mpox [29].

### Study Settings and Populations

#### Italy

Between 2022 and 2023, Italy reported approximately 955 confirmed cases of mpox [30], as part of the larger global 2022 outbreak. The majority (98%) of these cases were gay, bisexual, and other men who have sex with men [30]. Italian health authorities, including the Istituto Superiore di Sanità, responded swiftly by implementing measures such as contact tracing, targeted vaccination campaigns, and public health advice. The vaccination campaign was targeting “all people at risk” and was provided for free with the provision of gratuity for the Italian health system [30]. By early 2023, the incidence of mpox had significantly declined in Italy, partly due to vaccination and behavioral change.

#### Nigeria

Nigeria has reported mpox since 1971 and had its largest outbreak in 2017, before the global 2022 pandemic. However, public awareness about mpox is low, particularly in rural areas—perpetuating stigma, underreporting of cases, and reluctance to seek medical care [31,32]. Vaccines are an effective prevention tool; however, they remain unavailable in most African countries [33,34]. A positive development is that Nigeria received a 10,000-dose Modified Vaccinia Ankara vaccine donation from the US government in August 2024 [35], and in September 2025, initiated a targeted hotspot vaccination campaign with WHO-prequalified Modified Vaccinia Ankara-Bavarian Nordic mpox vaccines from Gavi, the Vaccine Alliance [36].

#### Thailand

In Thailand, the mpox outbreak also predominantly affects male individuals (97%), particularly gay, bisexual, and other men who have sex with men, many of whom also live with HIV [37,38]. The urgency of addressing mpox is underscored by Thailand’s approval of an mpox vaccination program, which commenced in March 2024 in Bangkok. This vaccine program was in the beginning limited to 1 health care provider (Red Cross Anonymous Clinic) [38], but it is now more widely available; however, it is still mostly in Bangkok and is prohibitively expensive for individuals, at between US $125 and US $485 (with an average monthly salary of around US $600) [39].

#### The United Kingdom

Between May 2022 and September 2023, there were over 3700 cases of mpox identified in the United Kingdom, peaking in July 2022 [40]. In total, 99% of cases in the United Kingdom were male; 96% identified as gay, bisexual, and other men who have sex with men [41]. No mpox-related deaths have been reported in the United Kingdom [42]. The monthly number of Clade IIb cases in England was between 1 and 41 in 2023-2025, compared to the peak of 1139 cases in July 2022 [43]. Public health responses to mpox in the United Kingdom included increasing public awareness, advice to self-isolate for 21 days, and contact tracing [44]. The UK mpox vaccination program started in June 2022, providing free vaccines to gay, bisexual, and other men who have sex with men considered to be at higher risk of infection, contacts of people with mpox, and people with potential occupational exposure.

Reflecting the differences in epidemiology between endemic and nonendemic settings, study populations of interest differ by country. In Nigeria, the focus is on children and pregnant and postpartum women, whereas in Thailand and the United Kingdom, it is on gay, bisexual, and other men who have sex with men—the population most affected in nonendemic countries. Both Nigerian and Thai studies will also recruit health care professionals. In Italy, methods are restricted to news media analysis, with no primary data collection.

### Data Collection

#### News Media (Italy, Nigeria, and the United Kingdom)

A systematic search of published news articles will be carried out in the 3 countries to explore representations of mpox in news media. Searches will be conducted of both online and in-print articles using keywords including “mpox,” “monkeypox,” “monkey pox,” and “MPX.” Publications will be selected using criteria such as scope of circulation and political alignment. The following newspapers will be included in the country analyses: *La Repubblica* and *Corriere della Sera* in Italy; *the Vanguard*, *the Nation*, *the Guardian*, *the* *Sun*, and *the Punch* in Nigeria; and *the* *Guardian*, *Sun*, *Times*, *Daily Mail*, and *Daily Mirror* in the United Kingdom.

#### Social Media (Nigeria, Thailand, and the United Kingdom)

We will conduct a systematic search of selected social media platforms to explore representations of mpox outbreaks and public discourse about mpox (including vaccination). Searches will use keywords such as “mpox,” “monkeypox,” “monkey pox,” and “MPX.” In Thailand, Thai-specific keywords such as 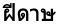 (*Phidard*), 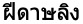 (*Phidardling)*, and 
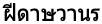
 (*Phidardwanon*) will be added. In the United Kingdom and Thailand, the analysis will focus on TikTok, whereas in Nigeria, it will focus on Instagram and Facebook. For these analyses, as content is shown to users algorithmically, user accounts will be created using the anticipated demographics of the populations of interest, and these accounts will be used only for research purposes. These platforms were selected for each country by popularity, access to data, and (for TikTok) methodological novelty. Multimodal data (including text, images, video, and sound) will be captured using standardized forms and analyzed thematically.

#### FGDs (Nigeria, Thailand, and the United Kingdom)

FGDs will be conducted to gain insights from participants about lived experiences of mpox [45], providing an in-depth understanding of participants’ knowledge and perceptions regarding the mpox outbreak. Topic guides will cover public perceptions, sources of information, attitudes toward transmission-reducing behaviors, public health messaging, and social and emotional impacts of mpox. FGD sampling will vary across settings depending on local mpox epidemiology (Table 2). The number of FGDs in each context will be determined by the number of different groups invited to participate, bearing in mind that 90% of themes are likely to be covered in 3-6 focus groups [46]. FGDs will comprise 3-10 participants each, depending on recruitment (which may be more or less challenging depending on stigma and other factors in local contexts).

**Table 2:** Populations of interest for VERDIQual^a^ interviews and focus group discussions.

Country	Period of mpox outbreak	Caregivers of people with mpox	People with mpox			Frontline health care workers (aged ≥18 years)
		Adult caregivers of children (aged ≤11 years)	Adolescents (aged 12-17 years)	Pregnant women and nursing mothers (aged ≥15 years)	Adults (aged ≥18 years)	
Nigeria	September 1, 2017^b^, to present	Yes	Yes	Yes	Yes, general population	Yes
Thailand	July 1, 2022, to present	No	No	No	Yes^c^	Yes
United Kingdom	May 1, 2022^d^, to present	No	No	No	Yes^e^	No

^a^VERDIQual: SARS-CoV-2 (and Mpox) Variants Evaluation in Pregnancy and Paediatrics Cohorts Qualitative Study.

^b^This date signifies the year of the largest mpox outbreak in Nigeria.

^c^Sexual and gender minorities, including gay, bisexual, and other men who have sex with men, and other people at high risk of mpox, including sex workers, people who use drugs, and people living with HIV.

^d^This date signifies the time point immediately preceding the first global mpox reports in Europe.

^e^Gay, bisexual, and other men who have sex with men.

In Nigeria, 10-15 in-person FGDs will be conducted with individuals who have acquired mpox (children and adolescents aged ≤17 years [or their caregivers where appropriate], pregnant or nursing women aged ≥15 years, and adults aged ≥18 years) and individuals aged ≥18 years affected by mpox (caregivers and health care workers). Recruitment will be supported by a community advisory board drawn from a local organization (Afrihealth Optonet Association), which will facilitate the identification, selection, and engagement of communities where mpox cases have been identified.

In Thailand, up to 8 in-person FGDs will be conducted with individuals who perceive themselves to be at risk of acquiring mpox (people who self-identify as gay, bisexual, and other men who have sex with men, transgender women, male and female sex workers, people who use drugs, and people living with HIV) and health care providers who provide care to patients with mpox, recruited from public health agencies and nongovernmental organizations (such as the Thailand Ministry of Public Health through the Department of Disease Control [Bamrasnaradura Infectious Diseases Institute and Bureau of Epidemiology], the Institute of HIV Research and Innovation, and the Hospital for Tropical Diseases). Recruitment will be supported by local nongovernmental organizations with strong community ties to the target populations. Specifically, male and female sex worker participants will be recruited with support from the Service Workers in Group Foundation; men who have sex with men who use drugs will be engaged through the Health and Opportunity Network; and men who have sex with men living with HIV will receive additional support from The Poz Home Thailand Foundation.

In the United Kingdom, 4-6 in-person or online [47] (participant choice) FGDs will be conducted with gay, bisexual, and other men who have sex with men who perceive themselves to have been at risk of mpox infection. Recruitment will be supported by voluntary sector organizations (George House Trust, NAZ Project London, and Positively UK) to ensure the inclusion of participants living with HIV, queer people of color, and people living outside of London.

#### SSIs (Nigeria and Thailand)

SSIs will be used to elicit rich information from key stakeholders’ experiences, perceptions, and opinions on mpox. We will continue to recruit participants until data saturation has been reached.

In Nigeria, SSIs will explore public perceptions, information sources, attitudes toward transmission-reducing behaviors, public health messaging, social and emotional impacts of mpox with a primary focus on the policies, and interventions for the management of mpox cases. One systematic review found that studies comprising between 9 and 17 interviews were likely to reach saturation [48]; however, this will vary depending on the diversity of participants and experiences included. Up to 20 SSIs will be conducted with (1) staff and policymaking officials from the Nigeria Centre for Disease Control and Prevention and State Ministries of Health; these are agencies involved in infectious disease surveillance and management, including mpox outbreaks; (2) key informants who have been diagnosed with mpox; and (3) health care workers.

In Thailand, SSI participants (aged ≥18 years) will be recruited from the national mpox surveillance database. Interviews will cover attitudes and experiences regarding mpox infection and associated stigmas, as well as policy and public health practice suggestions. Interviews will be discontinued if it is felt that saturation has been reached. Up to 20 SSIs will be conducted with individuals who self-identify as gay, bisexual, and other men who have sex with men, transgender women or *kathoey*, and who have had an mpox diagnosis. SSIs will be conducted among individuals who have experienced mpox either historically (eg, since the start of the Clade IIb outbreak) or more recently (within the past 4 weeks).

#### Participatory Photography (Thailand)

In Thailand, up to 10 SSI participants aged 18 years and older who provide additional consent will be enrolled in a participatory photography study (adapted from the photovoice approach) [49], following completion of their SSIs. This sample size is consistent with participatory photography in other health fields [50-53]. This study will involve participants providing photos and accompanying brief descriptions reflecting their lived experience, needs, values, and priorities in response to an mpox diagnosis. Researchers will ask participants to respond to twice-weekly prompts through SMS text message or email (depending on participant preference). Participants will be asked to submit up to 5 photographs to a secure university database in response to each prompt; they will also be invited to join 2 researcher-facilitated online workshops to share and discuss their photographs with each other. Captions and notes taken during workshops will be analyzed thematically and triangulated with SSI data.

### Data Management Plan

Data to be collected include qualitative text, images, and reports from news and social media; FGD and SSI audio and transcripts (with audio deleted after accuracy checking); rapid assessment procedure (RAP) sheets; and digital photographs (Thailand only). All data will be stored on a Penta Foundation password-protected shared drive in Italy (news media only), a password-protected project-based cloud storage database in Nigeria, and secure university shared drives in Thailand and the United Kingdom. Only VERDIQual study team members will have access to study data in each country; all staff working on this study have completed up-to-date training in research ethics and information governance.

### Team-Based Collaboration

This is a complex study involving multiple qualitative methods across 4 countries. We have established structures and processes to support team-based collaboration, facilitating methodological consistency and rigor. All teams have received standardized training in methods and analysis and use standardized protocols and standard operating procedures in the design and conduct of studies at their site, adapting them for their unique contexts.

### Data Analysis

#### Rapid Analysis

We will adopt an approach developed by the Rapid Research Evaluation and Appraisal Lab, which allows for simultaneous collection, synthesis, and reporting of large amounts of qualitative data in a short time using RAP sheets (Multimedia Appendix 2) [54]. The RAP sheet is a 2-column data collection and analysis form that (1) facilitates data standardization across study sites over time, (2) is adaptable to rapid changes in study designs and circumstances, (3) triangulates findings from different data sources, and (4) quickly synthesizes the most important findings [54]. Team members will capture key findings separately from SSIs, FGDs, news and social media analysis, and photovoice, with each row relating to a different SSI, FGD, or photovoice question or theme (in the case of news and social media analysis). There will be separate RAP sheets by method and site, as well as potentially by researcher. RAP sheets will record reflections per method across all participants (ie, no separate RAP sheets for each participant). RAP sheets will not replace in-depth thematic analysis and coding. They will allow us to generate early insights within and across countries, followed by in-depth analysis using an appropriate approach such as reflexive thematic analysis, while allowing us as researchers to provide feedback to key stakeholders at speed, an important consideration during an outbreak.

RAP sheets will also be used to guide collaborative analysis across countries. Through discussion and review of RAP sheets, we will reach consensus on cross-cutting themes as well as areas of difference. This will inform further in-depth coding and analysis of data from all countries. Initial findings from the rapid and thematic analyses of each dataset will be presented to community partners to allow for member checking.

#### News Media

News media articles will be analyzed via content analysis at each country level using NVivo qualitative data analysis software (version 14; QSR International Pty Ltd). A preliminary analysis of the Italian news media will be carried out on a selection of newspapers. All articles on mpox published in the selected newspapers between May 1, 2022, and December 31, 2022, will be included.

In Nigeria, all articles on mpox published during the peak periods of mpox outbreaks between May 2017 and December 2023 will be randomly selected from selected media houses. These articles will be reviewed by 2 independent reviewers to identify relevant themes and concepts central to the investigation and generate a codebook for analysis. Dual coding will be conducted until consensus is reached, and all coding will be conducted by 2 researchers.

The UK team will conduct a content analysis of selected articles that were published between May 1 and December 31, 2022 (the rise, peak, and decline of mpox in this context). The UK sample will comprise all relevant articles from the selected sources, published on a single day of the week, following a rotating weekday sampling schedule. Similar to Nigeria, a codebook will be developed by 2 researchers, and dual coding will be conducted until consensus is reached. The remainder of the coding will be carried out by an individual researcher (ST).

Themes identified from the analyses in Italy, Nigeria, and the United Kingdom will be reviewed by at least 2 researchers from each team to identify relevant cross-cutting themes of interest for the comparative analysis. Codes for these themes will be shared by the research teams and compared to identify commonalities and points of departure. The cross-country analysis will be led by the Italian team, with regular discussion involving the Nigerian and UK teams.

#### Social Media Analysis

Across the implementing countries, analysis of relevant mpox data from the selected social media platforms will be analyzed using NVivo (version 14) software to identify common themes. Three members of the Nigerian team (CO-A, EES-A, and GIA) will conduct discourse analysis of videos and posts on Facebook and Instagram using the hashtags “monkeypox,” “monkey pox,” and “mpox” in the search engine. Relevant mpox content posted from or on Nigeria from May 2017 to December 2023 will be extracted, and duplicates will be excluded. The structure and language of this content will be analyzed to explore representations of mpox, framing of responsibility, and perception of and attitudes to mpox vaccination.

At least 2 members from each of the UK and Thai teams will conduct an analysis of TikTok, a social media platform for sharing short videos. This will involve searching for videos with the search terms “monkeypox,” “monkey pox,” and “mpox” (Inline graphic 6. [*Phidard*], Inline graphic 7. [*Phidardling*], and Inline graphic 8. [*Phidardwanon*], respectively). Relevant TikToks will be selected, multimodal data extracted using a standardized form, and data analyzed thematically.

#### FGDs and SSIs

All FGDs and SSI audio will also be transcribed to facilitate subsequent in-depth thematic analysis using NVivo (version 14) software. Following accuracy checks, we will analyze data using the reflexive thematic analysis approach described by Braun and Clarke [55], which involves (1) data familiarization through repeated reading and note-taking; (2) coding sections of the transcript using a combination of a priori codes developed from literature review and review of RAP sheets, as well as inductive coding; (3) generating and refining themes to develop an overarching interpretation of the data; and (4) reflecting on researcher positionality throughout and how this may shape the analysis and interpretation. For all countries collecting these data, a proportion of FGD and SSI transcripts will be dual-coded, with findings compared and consensus reached for the codebook for each country.

#### Participatory Photography

This will only be undertaken in Thailand. Interpretation of visual materials and writing tasks will include a focus on the subject matter of separate photographs (eg, how many photographs are intended to document stigma or symptoms) as well as the visual narrative created by each participant (ie, how the visual data tells the story of each participant’s individual experience). Workshops will be cofacilitated by at least 2 researchers, allowing detailed notes to be kept and key themes to be identified between the researchers.

### Collaborative Analysis

To harness the full potential of this rich dataset comprising data obtained from 4 countries using a variety of methods, we will use a standardized approach for data integration (Table 3). This will occur at the interpretation stage. Teams in each country will complete RAP sheets in English (the project’s working language). This will facilitate collaboration as described earlier. Further in-depth analyses will be conducted by native speakers in the original language, with summaries of findings and interpretations completed in English. These summaries will be recorded in a multimethod data integration matrix, which will allow visualization of data by objective, method, and site, aiding synthesis across datasets. We acknowledge that some meaning will be lost as a result of language differences, but this pragmatic approach, which will include native speakers at all stages, allows us to integrate data while preserving context-specific meaning. The use of NVivo (including researcher memos) and preservation of RAP sheets and data integration matrices will ensure transparency by creating an audit trail.

**Table 3:** Example of multimethod data integration matrix.

	Study 1 (eg, FGDs^a^) findings	Study 2 (eg, SSI^b^) findings	Study 3 (eg, photovoice) findings	Study 4 (eg, TikTok) findings	Study 5 (eg, news media) findings	Synthesis (by objective across sites and methods)
State objective 1	Site 1 Site 2 Site n	Site 1 Site 2 Site n	Site 1 Site 2 Site n	Site 1 Site 2 Site n	Site 1 Site 2 Site n	Record synthesis here
State objective 2	Site 1 Site 2 Site n	Site 1 Site 2 Site n	Site 1 Site 2 Site n	Site 1 Site 2 Site n	Site 1 Site 2 Site n	Record synthesis here
State objective n	Site 1 Site 2 Site n	Site 1 Site 2 Site n	Site 1 Site 2 Site n	Site 1 Site 2 Site n	Site 1 Site 2 Site n	Record synthesis here
Synthesis (by method across sites)	Record synthesis here	Record synthesis here	Record synthesis here	Record synthesis here	Record synthesis here	Record synthesis here

^a^FGD: focus group discussion.

^b^SSI: semistructured interview.

### Patient and Public Involvement

Each country team has identified community partners who will lead the formation of CABs, comprising representatives of diverse patients, gatekeepers, and community member groups. CAB members will review study documents, contribute to analyses and manuscripts, and inform dissemination and future pandemic preparedness research priorities. In addition to regular meetings with their individual country teams, the CABs from Italy, Nigeria, Thailand, and the United Kingdom will have quarterly international CAB meetings to discuss findings, challenges, and lessons learned.

### Ethical Considerations

This study has received ethics approval from the following institutional review boards: in Nigeria, from the National Health Research Ethics Committee (NHREC/01/01/2007); in the United Kingdom, from University College London (6698/005); and in Thailand, from the Mahidol University, Faculty of Tropical Medicine Ethical Committee (MUTM 2025-001-01). Ethical review and approval are not indicated for the news media analysis in Italy. Participant consent is not applicable or required for data sourced from news and social media, as this information is considered public (if accounts are openly accessible). However, for social media analyses, usernames will not be included in reports, and quotes will only be used where they are not considered to be sensitive (ie, talking about traumatic experiences). Ethics around consent and anonymity in social media research is a constantly evolving issue, and special care will be taken [56]. Given the potentially delicate and emotive nature of the research, participants will be informed that they are not obliged to answer questions they find difficult. Written informed or verbal consent will be sought from individuals recruited for FGDs, SSIs, and participatory photography in each country where it applies and will be obtained by members of the research team. In addition to written or verbal consent, for participatory photography in Thailand, emphasis will be placed on visual anonymity, with participants advised to avoid capturing identifiable faces or settings that could compromise privacy or safety. To ensure that the consent process accommodates the needs of study participants (which include adolescents, people living with HIV, and gay, bisexual, and other men who have sex with men), consent procedures will prioritize privacy and autonomy. In Nigeria, written assent and parental permission will be sought for adolescent participants aged 12 to 17 years in accordance with national guidelines [57]. Country teams include experienced qualitative researchers with expertise in working with young people and in HIV and sexual health settings. Participants will be given sufficient time to review the participant’s information sheet, ask questions, and discuss their concerns. All qualitative data will be anonymized; identifying information will be removed during transcription and analysis. For participatory photography in Thailand, participants will maintain ownership and control over their photographs, with the sole decision of which images to be shared, discussed, or included in the dissemination materials. All approved images will be reviewed collaboratively to ensure alignment with participants’ intent while adhering to ethical standards of respect and confidentiality. All participants were informed that participation is voluntary and that refusal or withdrawal from the study will not affect access to any services, including those provided by community partners. Participant reimbursement followed local ethics guidelines. In Nigeria, participants received approximately US $4 for time and transport; this sum was approved by the National Health Research Ethics Committee. In Thailand, participants received US $15 as reimbursement for their time and transport; this sum was approved by the Mahidol University Faculty of Tropical Medicine Ethical Committee. In the United Kingdom, FGD participants received US $26 shopping vouchers, which was approved by the University College London Research Ethics Committee.

## Results

### Study Status

At the time of writing, all teams—Italy, Nigeria, the United Kingdom, and Thailand—have obtained the required national and institutional ethics approvals. The study was funded in September 2023, and initial institutional review board approvals were received in each country on varying dates: in Nigeria, January 2024; the United Kingdom, August 2023; and Thailand, January 2025. The development of data collection tools including FGD and SSI topic guides has been completed in collaboration with the CABs. Recruitment has commenced in all countries, with the completion of all qualitative data collection anticipated by October 31, 2025. The VERDIQual umbrella (master) protocol and multiple relevant study tools are available online at protocols.io [58]. At the time of publication, Italy has completed news media data collection and is currently conducting analysis; the Nigerian and Thai teams have completed data collection for all components of their qualitative studies, and analysis is ongoing; the United Kingdom team has finished all FGDs and is analyzing their data. The earliest results manuscripts are expected to be published between May and July 2026.

### Results Dissemination Plan

A comprehensive, implementation science–guided dissemination plan [59] to share VERDIQual findings is being codeveloped and will be coimplemented with the international CAB to reach diverse stakeholders and maximize impact on pandemic preparedness in study countries and globally. Dissemination is planned for 3 main audiences:

- Funder: VERDIQual reports to the European Commission.
- Scientific: We will reach the scientific community through abstracts and invited presentations at major national, regional, and international conferences and publications in peer-reviewed journals.
- Nonscientific or community: The nonscientific audience has been segmented into patients or patient advocacy groups; the general public or communities; providers, including health care workers and health professional organizations; and policymakers such as government officials, Ministries of Health, and other relevant policymaking agencies.

The mode of dissemination for each nonscientific or community audience will be determined by each country team for their local setting. A range of options include social media messaging for patients; community town hall meetings for study communities; results communications through flyers at health facilities; and technical reports, policy briefs, and presentations at policymaking meetings for policymakers.

## Discussion

### Overview

The mpox outbreak continues to be a pressing public health concern. The experience of the 2019 SARS-CoV-2 pandemic allowed lessons to be learned and insights into missed opportunities for application to the 2022 mpox pandemic. Beyond the public health experience of past pandemics, rigorous data on the experiences of individuals affected by mpox will be critical to better inform public health interventions and messaging for mpox and future pandemics.

### Expectations for Principal Findings

Given our inclusion of 3 global regions, mpox endemic and nonendemic countries, general and marginalized populations, and high- and low-resource settings, we expect our principal findings to be applicable to a range of settings. Furthermore, our application of intersectionality theory will also facilitate considerations for intersecting identities and characteristics in equity-centered pandemic responses. By this, we mean intentionality in pandemic preparedness, inclusive of people with 1 or more stigmatized and vulnerable characteristics or identities with respect to infection or access to health interventions, such as geographical location, gender, pregnancy, young age, sexual orientation, race, and class or economic status. Our approach to dissemination and potential translation of our findings into policy are described in our dissemination plan mentioned earlier.

### Comparison to Prior Work

Several qualitative studies have documented the experiences of people with mpox, including people with HIV coinfection, during the 2022 pandemic [22-25,60-64]. However, almost all of these studies were conducted among gay, bisexual, and other men who have sex with men in nonendemic, high-income countries such as France, the United States, the United Kingdom, Australia, and China. Notable studies from Africa include 2 studies among men who have sex with men and the general population in Nigeria [65,66]. At the time of writing, there is a paucity of studies on the experiences of different patient populations with mpox in endemic African countries or across both endemic and nonendemic settings.

### Anticipated Challenges and Mitigation Strategies

For a multicountry, multimethod study such as VERDIQual, implementation challenges may occur due to variations in public health infrastructure, case burden, and the range of populations disproportionately affected by mpox. Language differences, legal and ethical regulations, cultural norms, and stigma, particularly around age, gender identity, and sexual orientation, may impact data collection. To mitigate these, we have adopted a country-led approach to the collaboration, allowing for the selection of locally feasible qualitative research methods, local adaptation of study tools, and application of locally appropriate recruitment strategies using CABs while providing standardized training and requiring standardized reporting for all country teams to ensure methodological consistency across sites.

## Appendix

Multimedia Appendix 1 COREQ checklist.

Multimedia Appendix 2 Example of rapid assessment procedure sheet to be used for rapid qualitative research data collection.

## Abbreviations

CAB: community advisory board
COREQ: Consolidated Criteria for Reporting Qualitative Research
FGD: focus group discussion
RAP: rapid assessment procedure
SSI: semistructured interview
VERDI: SARS-CoV-2 (and Mpox) Variants Evaluation in Pregnancy and Paediatrics Cohorts
VERDIQual: SARS-CoV-2 (and Mpox) Variants Evaluation in Pregnancy and Paediatrics Cohorts Qualitative Study
WHO: World Health Organization
