# Supplementary material for: Rapid Qualitative Approaches in Pandemic Research: Protocol for an Exploratory Qualitative Multimethod Study (VERDIQual) on Mpox in Italy, Nigeria, Thailand, and the United Kingdom
Source: JMIR Res Protoc. 2026 Jan 15;15:e77321. doi: 10.2196/77321 (PMC12856405; doi:10.2196/77321)
Supplement: Multimedia Appendix 1 [file resprot_v15i1e77321_app1.pdf]

Consolidated criteria for reporting qualitative studies (COREQ): 32-item checklist

For further information about the COREQ guidelines, please see Tong *et al.*, 2017: https://doi.org/10.1093/intqhc/mzm042

No.	Item	Description	Section #
**Domain 1: Research team and reflexivity**			
The multidisciplinary research team possesses diverse expertise essential for this study, including infectious diseases (CO, MLP, EJN, DB, TEG, EES, GIA, CA, AAA, KY, TCW, WW, NS, TM, SD, WG, ST, NASA), qualitative methodology (CO, MLP, EJN, DB, EES, GIA, WW, TM, SD, ST, NASA), public health (CO, EES, GIA, CA, AAA, KY, TCW, TM, UZ, ST, NASA), implementation science (CO, CA, NASA), sociology (EJN, DB, TCW, TM), sexual health (TEW, TCW, WW, NS, ST, NASA), and psychology (SD).			
Personal characteristics			
1.	Interviewer/ facilitator	Which author/s conducted the interview or focus group?	CO, MLP, EJN, TCW, EES and GIA will conduct interviews and focus group discussions in their respective countries.
2.	Credentials	What were the researcher's credentials? *E.g. PhD, MD*	CO: MPH<br>MLP: MSc<br>EJN: PhD<br>DB: DPhil<br>TEG: PhD<br>EES: MPH<br>GIA: MPH<br>CA: MPH<br>AAA:MD<br>KY:MD<br>TCW: PhD<br>WW: MA<br>NS: MA<br>TM: PhD<br>SD: PhD<br>WG: MA<br>UA: PhD<br>ST: MD, PhD, Consultant in HIV/Sexual Health<br>NASA: MD, Professor of Pediatrics
3.	Occupation	What was their occupation at the time of the study?	CO: Training Program Coordinator<br>MLP: Research Fellow<br>EJN: Research Fellow<br>DB: Research Fellow<br>TEG: Associate Professor<br>EES: Research Assistant<br>GIA: Research Assistant<br>CA: Training Program Coordinator<br>AAA: Research Assistant<br>KY: Research Assistant<br>TCW: Research Fellow<br>WW: Senior Researcher<br>NS: Senior Researcher<br>TM: Research Fellow<br>SD: Research Fellow

			WG: Patient Advocate and Activist<br>UA: Health Systems Consultant<br>ST: Consultant in HIV/sexual health<br>NASA: Senior Technical Advisor for Pediatric & Adolescent in HIV and Professor of Pediatrics
4.	Gender	Was the researcher male or female?	11 of 19 authors are female<br>CO: Female<br>MLP: Female<br>EJN: Female<br>DB: Male<br>TEG: Male<br>EES: Female<br>GIA: Female<br>CA: Male<br>AAA: Female<br>KY: Female<br>TCW: Male<br>WW: Female<br>NS: Male<br>TM: Male<br>SD: Female<br>WG: Male<br>UA: Male<br>ST: Female<br>NASA: Female
5.	Experience and training	What experience or training did the researcher have?	In addition to the authors' prior experience and training before the start of this study, all authors participated in a training program provided by the Rapid Research Evaluation and Appraisal Lab (RREAL). This training focused on conducting qualitative research and utilizing Rapid Assessment Procedures (RAP) sheets for qualitative data collection and analysis.
Relationship with participants			
6.	Relationship established	Was a relationship established prior to study commencement?	Study teams were selected for this consortium based on their strong ongoing relationships with mpox-affected communities. Furthermore, every country team has a highly engaged Community Advisory Board that will strengthen and expand relationships with affected communities in each country.
7.	Participant knowledge of the interviewer	What did the participants know about the researcher? *E.g. Personal goals, reasons for doing the research*	Participants will be informed about the researchers through a Participant Information Sheet, which provides background on the study, details about the researchers involved, their professional roles, and the organizations they represent. Prior to the commencement of any interviews or FGDs interviewers will introduce themselves to participants will the following information already captured in the participant information sheet.

8.	Interviewer characteristics	What characteristics were reported about the interviewer/facilitator? *E.g. Bias, assumptions, reasons and interests in the research topic*	Not applicable as this is a protocol paper
**Domain 2: Study design**			
Theoretical framework			
9.	Methodological orientation and theory	What methodological orientation was stated to underpin the study? *E.g. grounded theory, discourse analysis, ethnography, phenomenology, content analysis*	An exploratory multi-method, multi-site qualitative study comprises content analysis of news and social media, focus group discussions (FGDs), semi-structured interviews (SSIs) and participatory photography. *See line 172 – 174, and Figure 1.*
Participant selection			
10.	Sampling	How were participants selected? *E.g. purposive, convenience, consecutive, snowball*	The study countries and sites were purposively selected based on the number of reported mpox cases and geographical diversity. Each site employed different qualitative approaches. The study sites included Italy, Nigeria, Thailand, and the UK. The approaches were as follows:<br>• **Print Media**: Media houses were selected based on their coverage, readership, and popularity.<br>• **Social Media**: Social media platforms were chosen for a combination of their popularity, data accessibility, and methodological novelty.<br>• **Focus Group Discussions (FGDs) and Semi-Structured Interviews (SSIs)**: FGD sampling varied across settings, depending on the local epidemiology of mpox.<br>*See line 230-268*<br>• **Participatory Photography**: This applies only to Thailand. Participants involved in SSIs will be included to provide photos.<br>*See line 375- 386, & Figure 1.*
11.	Method of approach	How were participants approached? *E.g. face-to-face, telephone, mail, email*	**Print Media & Social Media**: N/A<br>**FGDs & SSIs**: Participant selection will be conducted by identified community organizations in each country, which serve as local Community Advisory Boards for this study. *See line 323-325, 336-341 & 345-347*
12.	Sample size	How many participants were in the study?	**Print Media & Social Media**: Articles and posts will be randomly selected from a pool of mpox-related content published during the peak of the mpox outbreak, which varies by country. *See line 430-470*<br>**FGDs & SSIs**: A total of 34 FGDs will be conducted across all study sites, with each FGD having a minimum of 3 and a maximum of 10 participants. Participants for SSIs will be selected based on

			specific characteristics or job roles relevant to the study. *See line 314-316, 318, 327, 343, 358-364 & 369-373*<br>**Participatory Photography:** This applies to only Thailand. A total of 10 participants who participated in the SSIs will take part in sharing photos. *See Line 376– 378.*
13.	i	How many people refused to participate or dropped out? What were the reasons for this?	Not applicable as this is a protocol paper
Setting			
14.	Setting of data collection	Where was the data collected? *E.g. home, clinic, workplace*	Data will be collected in private rooms at health facilities and in community spaces acceptable to participants.
15.	Presence of non-participants	Was anyone else present besides the participants and researchers?	Not applicable as this is a protocol paper, however, interviews are designed to involve only participants and researchers.

16.	Description of sample	What are the important characteristics of the sample? *E.g. demographic data, date*	**Print and social media**: data will encompass text, images, video, and audio content but no direct contact with human subjects. *See Line 284 -304.*<br>**FGDs/SSIs:** Participants will be selected to reflect differences in mpox epidemiology between endemic and non-endemic settings, with study populations varying by country. In Nigeria, the focus will be on children, pregnant, and postpartum women, while in Thailand and the UK, the focus is on gay, bisexual, and other men who have sex with men (GBMSM). Additionally, healthcare professionals and policymakers will be recruited in both Nigeria and Thailand. *See Line 270-275 & Table 1.*<br>**Participatory Photography:** This applies to only Thailand. Participants who participated in the SSIs will take part in sharing photos. *See Line 379-381*
Data collection			
17.	Interview guide	Were questions, prompts, guides provided by the authors? Was it pilot tested?	**Print Media & Social Media**: N/A<br>**FGDs/SSIs:** guides will be developed by each country's research team, focusing on topics such as public perceptions, information sources, attitudes toward transmission-reducing behaviors, public health messaging, and the social and emotional impacts of mpox.<br>*See Line 309 - 311.*<br>**Participatory Photography**: This applies to

			only Thailand. Participants will respond to prompts provided by researchers based on the type of pictures shared by the participants. *See line 381 – 385.*
18.	Repeat interviews	Were repeat interviews carried out? If yes, how many?	Not applicable as this is a protocol paper
19.	Audio/visual recording	Did the research use audio or visual recording to collect the data?	**Print Media & Social Media**: N/A<br>**FGDs/SSIs:** audio recordings or images will be used based on the study country and planned study design ie qualitative interviews vs photovoice. *See Line 390-392 and Figure 1.*<br>**Participatory Photography:** This applies to only Thailand. Captions and notes written by researchers during the interviews will be used**.** *See Line 385-386*
20.	Field notes	Were field notes made during and/or after the interview or focus group?	Field notes will be made during and completed after the interviews and focus groups, according to the rapid qualitative analysis approach. *See Lines 406-418.*
21.	Duration	What was the duration of the interviews or focus group?	Not applicable as this is a protocol paper
22.	Data saturation	Was data saturation discussed?	Given the rapid qualitative analysis approach and study design, data saturation is not expected to be a key consideration
23.	Transcripts returned	Were transcripts returned to participants for comment and/or correction?	Given the rapid qualitative analysis approach, we do not plan to return transcripts to participants for comment
**Domain 3: analysis and findings**			
Data analysis			
24.	Number of data coders	How many data coders coded the data?	**Print Media & Social Media:** a minimum of two coders per country will identify relevant themes and concepts central to the investigation, thereby generating a codebook for analysis. *See Line 438-440.*<br>**FGDs/SSI:** while rapid assessment procedure (RAP) will be employed for the FGDs/SSIs, with RAP sheets used to collect and analyze data in real time (See line 406-422) and a further in-depth thematic analysis using Nvivo software will be conducted. *See line 473-483*<br>**Participatory photography:** This applies to only Thailand**.** A minimum of two researchers will be involved in taking notes during this process<br>*See line 486 - 491.*
25.	Description of the coding tree	Did authors provide a description of the coding tree?	Not applicable as this is a protocol paper

26.	Derivation of themes	Were themes identified in advance or derived from the data?	**Print Media & Social Media:** themes will be derived deductively from all selected articles and content across the various social media platforms. *See Line 437 - 470.*<br>**FGDs/SSIs:** themes will be generated during data collection using RAP sheets., however further in-depth coding and analysis will be done using Nvivo.<br>*See Line 473 – 481 and 424-426*<br>**Participatory photography:** N/A
27.	Software	What software, if applicable, was used to manage the data?	**Print Media & Social Media:** NVivo software will be used for data analysis. *See Line 431 and 457-458.*<br>**FGDs/SSIs**: We will adopt an approach developed by the Rapid Research Evaluation and Appraisal Lab (RREAL), which enables a rapid manual simultaneous collection, synthesis, and reporting of large qualitative datasets, using Rapid Assessment Procedures (RAP) sheets, *See Line 407-422* and the Nvivo software for indepth thematic analysis.<br>*See line 457, 473-474*
28.	Participant checking	Did participants provide feedback on the findings?	Given the rapid qualitative analysis approach, we do not plan to conduct participant checking
Reporting			
29.	Quotations presented	Were participant quotations presented to illustrate the themes / findings? Was each quotation identified? *E.g. Participant number*	This is a protocol paper; however, when we publish results, participant quotations will be used to elucidate identified themes, with participant numbers.
30.	Data and findings consistent	Was there consistency between the data presented and the findings?	Not applicable as this is a protocol paper
31.	Clarity of major themes	Were major themes clearly presented in the findings?	Not applicable as this is a protocol paper
32.	Clarity of minor themes	Is there a description of diverse cases or discussion of minor themes?	Not applicable as this is a protocol paper

Developed from: Allison Tong, Peter Sainsbury, Jonathan Craig, Consolidated criteria for reporting qualitative research (COREQ): a 32-item checklist for interviews and focus groups, International Journal for Quality in Health Care, Volume 19, Issue 6, December 2007, Pages 349–357, https://doi.org/10.1093/intqhc/mzm042
