# Supplementary material for: Rapid Qualitative Approaches in Pandemic Research: Protocol for an Exploratory Qualitative Multimethod Study (VERDIQual) on Mpox in Italy, Nigeria, Thailand, and the United Kingdom
Source: JMIR Res Protoc. 2026 Jan 15;15:e77321. doi: 10.2196/77321 (PMC12856405; doi:10.2196/77321)
Supplement: Multimedia Appendix 2 [file resprot_v15i1e77321_app2.pdf]

Focus Group Discussion RAP Sheet

**Project Name:** SARS-coV2 variants Evaluation in pRegnancy and peDIatrics cohorts (VERDI) **Date of Interview:**<br>**Participant Category:**<br>**Data Source:** (Focus Group Discussion or Semi-structured Interview)<br>**Researcher name(s):**

Instructions: *If there is a third person facilitating, they may take notes on the RAP sheet during discussion. Otherwise, RAP sheet is completed the following morning (or straight after if appropriate) with all facilitators. The same RAP sheet is used by all facilitators for all interviews (focus groups, individual semi-structured interview). Interviews will be transcribed which will include all of the discussion. These RAP sheets will summarise main points of interest as agreed by the facilitators, as well as notes on the dynamic, and pertinent quotes as appropriate.*

**Question**	**Discussion (could include quotes)**<br>*This is the researcher's direct summary of what is being said, including selected verbatim representative quotes made by participants.*	**Dynamic**<br>*Researcher's observations of Interactions between participants, including verbal and non-verbal. This could be highlighting areas of dissent, agreement or discomfort.*	**Discussion/thoughts/ideas**<br>*This is the researcher's reflections on <u>data from across the FGDs</u>, allowing them to synthesise findings rapidly and progress analysis.*
Can you write down on a post-it note, 3 words you think of when you hear the word monkeypox/ mpox.			
Do you remember when you first heard about mpox?			
Do you remember trying to find information about it?			
What did you find out about how it was transmitted and what do you think about this?			
What do you think about the way mpox was discussed by these different sources?			

Focus Group Discussion RAP Sheet

What could be done differently?			
How important was it to you to avoid mpox?			
What are your thoughts on the mpox vaccine?			
Activity: Prevention<br>What other ways of preventing mpox were you made aware of?			
How were your experiences of this outbreak compared to COVID			
How about other infections – how does mpox compare?			
How did you find our discussion today?			
